# Proton pump inhibitors and the risk of urolithiasis: A Mendelian randomization study

**DOI:** 10.1097/MD.0000000000045646

**Published:** 2025-11-21

**Authors:** Jiawei Guo, Xinyu Chen, Yongqi Dou, Yongjiang Xiong, Tao Zhao

**Affiliations:** a Department of Urology, Yongchuan Hospital of Chongqing Medical University, Chongqing, PR China.

**Keywords:** causal association, genome-wide association study, Mendelian randomization, proton pump inhibitors, urolithiasis

## Abstract

Observational studies suggest that proton pump inhibitors (PPIs) increase the risk of urolithiasis (UL). However, the causal correlation between the 2 is still unclear. To examine the causal relationship between PPIs and UL, this study used 2-sample Mendelian randomization. The genome-wide association studies (GWAS) of Omeprazole, Esomeprazole, Lansoprazole, and Rabeprazole medication can be obtained from GWAS catalog. Furthermore, we acquired UL-related single nucleotide polymorphisms from the integrative epidemiology unit OpenGWAS. To access the causality of PPIs and UL, we performed a 2-sample Mendelian randomization. The primary approach to analysis was random-effects inverse variance weighting. In addition, we also used another dataset of UL to further verify the causal role. We discovered that there was a 5% increase in the incidence of UL in the use of Rabeprazole medication (test group: odd ratio [OR] = 1.053, 95% confidence interval [CI]: 1.005–1.103; validation group: OR = 1.051, 95% CI: 1.003–1.101). Furthermore, we found that Esomeprazole was negatively correlated with UL in the inverse variance weighting method (test group: OR = 0.914, 95% CI: 0.841–0.993; validation group: OR = 0.943, 95% CI: 0.890–0.999). The results of sensitivity analyses supported our conclusion. In conclusion, our results suggest a causal relationship of PPIs to UL. For patients requiring antiacid therapy, esomeprazole may be a better option than Rabeprazole, especially in patients with high-risk factors for urolithiasis.

## 1. Introduction

Urolithiasis (UL) is one of the most common diseases in urology, affecting about 15% of the world’s population.^[1,2]^ In addition, the prevalence of UL is rising, which puts a heavy financial and social strain on healthcare.^[3,4]^ It is therefore important to figure out the risk factors of UL and discover the relationship between them.

Proton pump inhibitors (PPIs) stand as a globally prescribed mainstay for treating a variety of gastric acid-related conditions. They are instrumental in alleviating the symptoms of gastroesophageal reflux disease (GERD), combating *Helicobacter pylori* infections, and promoting the healing of gastric ulcers.^[5]^ However, with the long-term use and misuse of PPIs, a series of adverse events have also occurred.^[6]^ Numerous studies have examined the impact of PPIs use on the risk of kidney stones.^[7–9]^ For instance, Sui et al found that the use of PPIs might decrease the levels of citrate and magnesium in urine, which could increase the risk of kidney stones, potentially weakening our body’s inhibitory effects on the formation of kidney stones.^[9]^ However, these studies lack information on the type of PPIs used. In addition, these studies were vulnerable to unmeasured confounding variables and reverse causation, which made it difficult to accurately determine the causative relationship between the 2 diseases.^[10]^ Mendelian randomization (MR) uses single nucleotide polymorphism (SNP) as a representative of exposure, which can minimize residual confusion in observational studies, thereby enhancing exposure and outcome causal inference.^[11,12]^ Thus, we conducted a 2-sample MR study to examine the genetic correlation between PPIs and UL.

## 2. Materials and methods

### 2.1. Study design

In the 2-sample MR analysis, SNPs used as the instrumental variants (IVs) should satisfy 3 essential criteria: They should have a significant correlation with exposure; They should be unaffected by confounding variables; They affect the outcome only through exposure.^[13]^ MR design can offer accurate causal effect estimates and control for any potential confounding factors only when all 3 requirements are satisfied, demonstrating the causal links between the 2.^[14]^

### 2.2. Genome-wide association studies (GWAS) summary data for PPIs and UL

All of the related data for SNPs are from the open GWAS. Table 1 displays the detailed GWAS data information. The GWAS of Omeprazole, Esomeprazole, Lansoprazole, and Rabeprazole medication can be obtained from GWAS catalog (https://www.ebi.ac.uk/gwas/home) (Omeprazole: GCST90042015; Esomeprazole: GCST90042487; Lansoprazole: GCST90041995; Rabeprazole: GCST90042442). The GWAS of UL can be obtained from MRC integrative epidemiology unit OpenGWAS (https://gwas.mrcieu.ac.uk/). No further ethical approval was required because all of the data used had already been made available in the public database.

**Table 1:** The detailed GWAS data information.

Trait	Ancestry	Year	Sample size	GWAS_ID
Omeprazole	European	2021	456,276 (26,869/429,407)	GCST90042015
Esomeprazole	European	2021	456,276 (1520/454,756)	GCST90042487
Lansoprazole	European	2021	456,276 (16,241/440,035)	GCST90041995
Rabeprazole	European	2021	456,276 (824/455,452)	GCST90042442
Urolithiasis (test)	European	2021	218,792 (5347/213,445)	finn-b-N14_UROLITHIASIS
Urolithiasis (validation)	European	2021	488,346 (6223/482,123)	GCST90018935

GWAS = genome-wide association studies.

### 2.3. Selection of genetic instrumental variables

To acquire a suitable number of SNPs in this study, we change the *P* value of PPIs to 5 × 10^‐6^.^[15,16]^ Then, in order to get independent instruments, relevant SNPs were clustered using linkage disequilibrium with *R*^2^ < 0.001 and cluster distance (kb) = 10,000. The instrumental variables’ strength was assessed using *F* statistics.^[17]^ The weak instrumental variable is defined by the threshold *F* < 10, so the deviation it causes can be disregarded. We harmonized the SNPs^[18]^ after using PhenoScanner^[19]^ to look for the relevant SNPs’ phenotypes, excluding any SNPs that might cause UL (such as body mass index, coronary artery disease, glycemic traits, serum urate, and calcium, etc).^[20–24]^ Palindrome SNPs were also removed in this process. Ultimately, the remaining SNPs were chosen as IVs for the subsequent MR examination.

### 2.4. Statistical analysis

We employed 5 methods, primarily inverse variance weighted (IVW) methods, to determine if PPIs and UL are causally related.^[25]^ The remaining 4 methods include: MR-Egger,^[26]^ weighted-median,^[27]^ weighted mode,^[28]^ and simple mode.^[29]^ The following guidelines are adhered to: When pleiotropy and heterogeneity are absent, IVW is the most dependable result to consider as a top priority; When merely heterogeneity exists, the result of the preferred weighted medium method and IVW (multiplicative random effects); The MR-Egger method’s calculations yield better results when multiple validity is present.^[30]^ In addition, a number of sensitivity studies were performed to assess the association’s strength. Initially, funnel plots and the Cochran *Q* test were used to evaluate heterogeneity.^[31]^ Second, we used MR-Egger regression to determine whether the intercept was statistically different from zero in order to identify the presence of directed pleiotropy. Third, we confirmed the robustness of the results by applying the leave-one-out approach.^[32]^ Fourth, we use the MR pleiotropy residual sum and outlier test to look for potential outliers.^[33]^ We presented the relationships between PPIs and the risk of UL using odds ratios (ORs) along with their 95% confidence intervals (CIs). With the aid of the related R packages “mrpresso1.0” and “TwoSampleMR0.5.8” and their dependent expansions, the aforementioned study is examined and displayed in R v.4.3.0. A statistically significant difference in this study was defined as *P* < .05. This investigation adheres to the Strengthening the Reporting of Observational Studies in Epidemiology guideline.

## 3. Results

**Figure 1.**
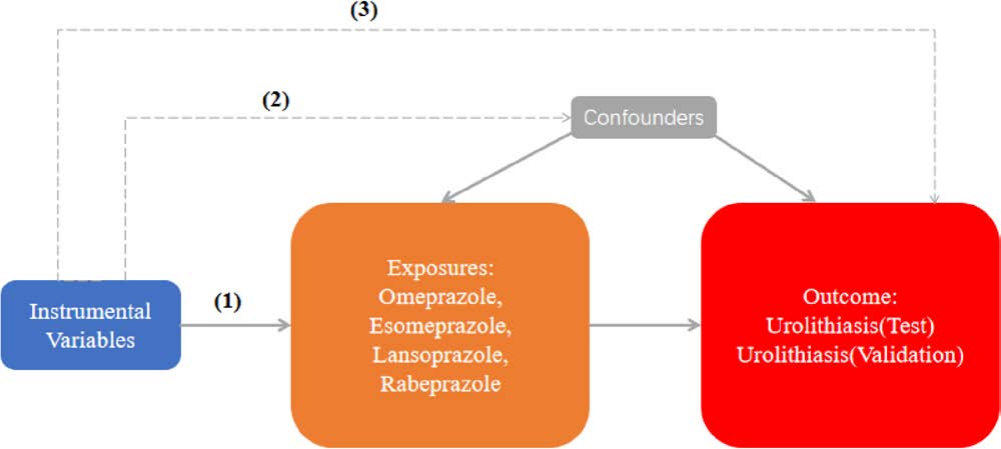
Study flow chart. (1) The IVs should have a significant correlation with exposure; (2) The IVs should be unaffected by confounding variables; (3) The IVs affect the outcome only through exposure. Dashed lines indicate no correlation, and solid lines indicate the correlation. IV = instrumental variables.

**Figure 2.**
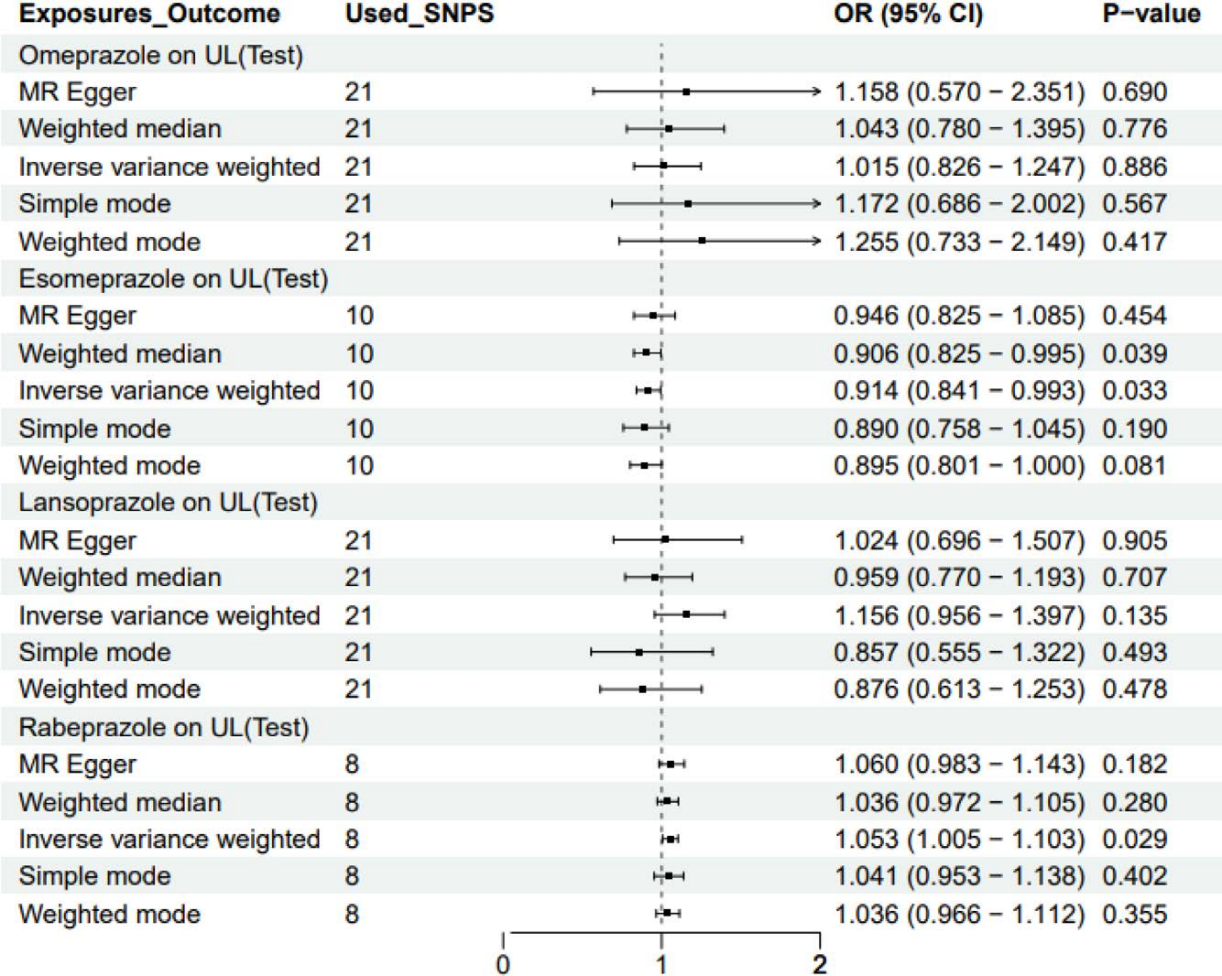
OR plot for PPIs on UL in test group. OR = odds ratio, UL = urolithiasis, PPIs = proton pump inhibitors.

**Figure 3.**
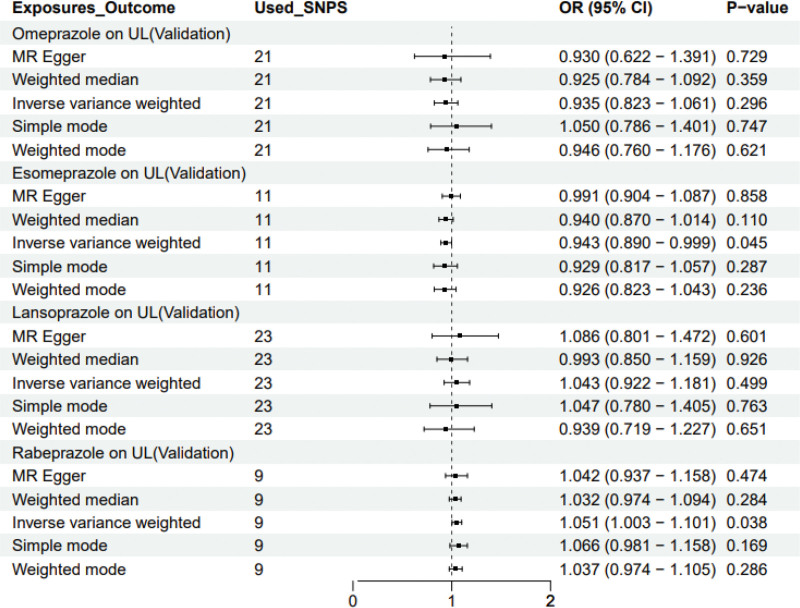
OR plot for PPIs on UL in validation group. OR = odds ratio, UL = urolithiasis, PPIs = proton pump inhibitors.

**Figure 4.**
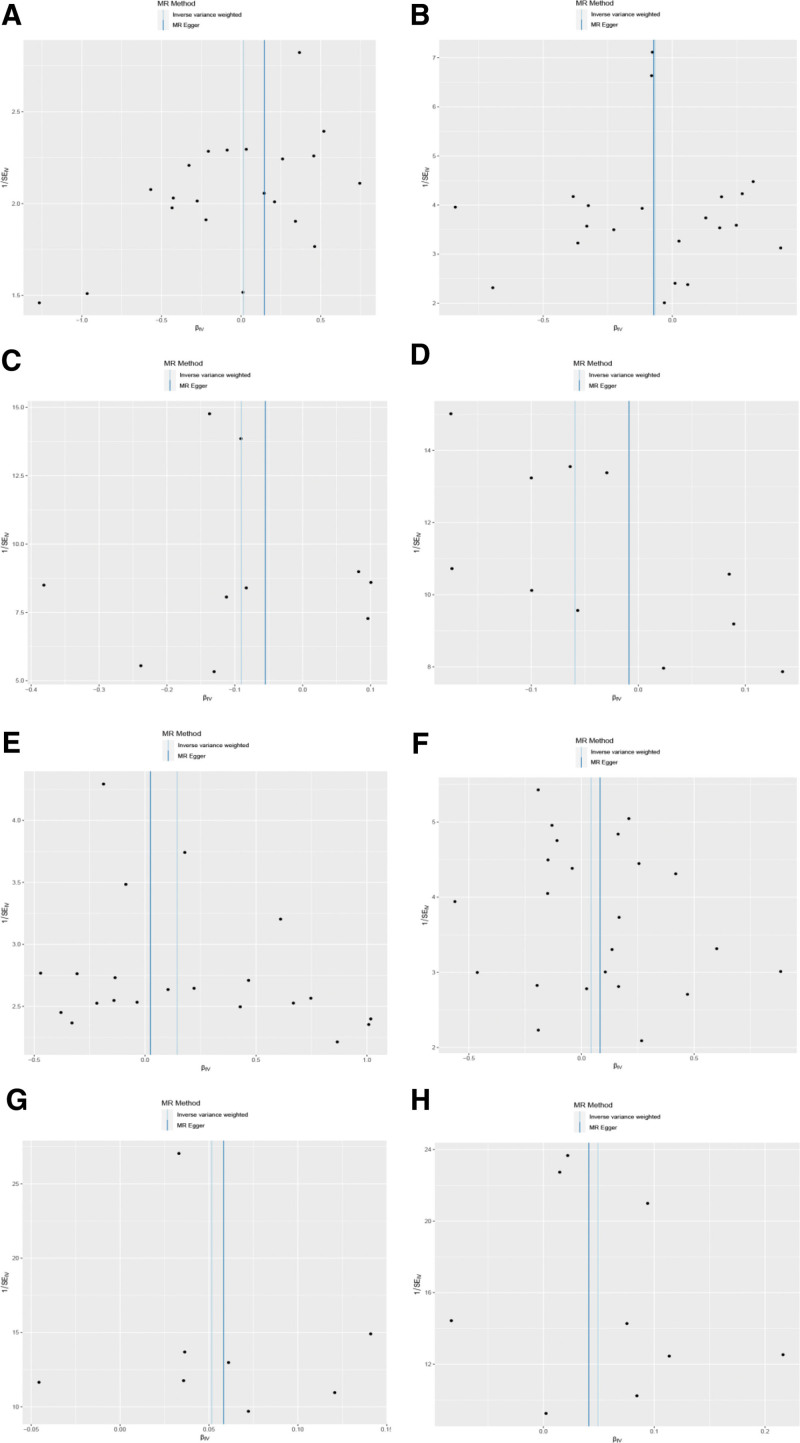
Funnel plot analysis: (A) Omeprazole–UL (test); (B) Omeprazole–UL (validation); (C) Esomeprazole–UL (test); (D) Esomeprazole–UL (validation); (E) Lansoprazole–UL (test); (F) Lansoprazole–UL (validation); (G) Rabeprazole–UL (test); (H) Rabeprazole–UL (validation). UL = urolithiasis.

**Figure 5.**
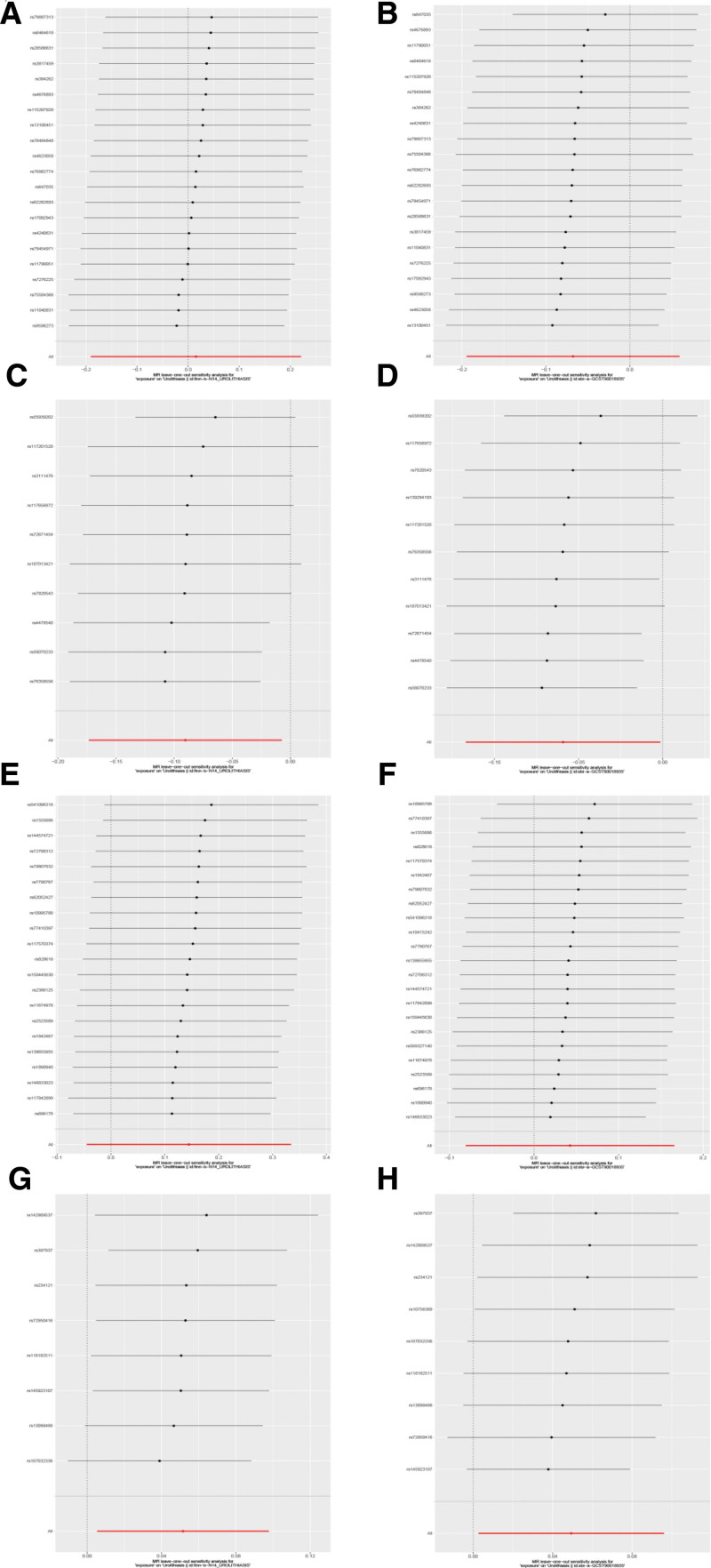
Leave-one-out analysis: (A) Omeprazole–UL (test); (B) Omeprazole–UL (validation); (C) Esomeprazole–UL (test); (D) Esomeprazole–UL (validation); (E) Lansoprazole–UL (test); (F) Lansoprazole–UL (validation); (G) Rabeprazole–UL (test); (H) Rabeprazole–UL (validation). UL = urolithiasis.

**Figure 6.**
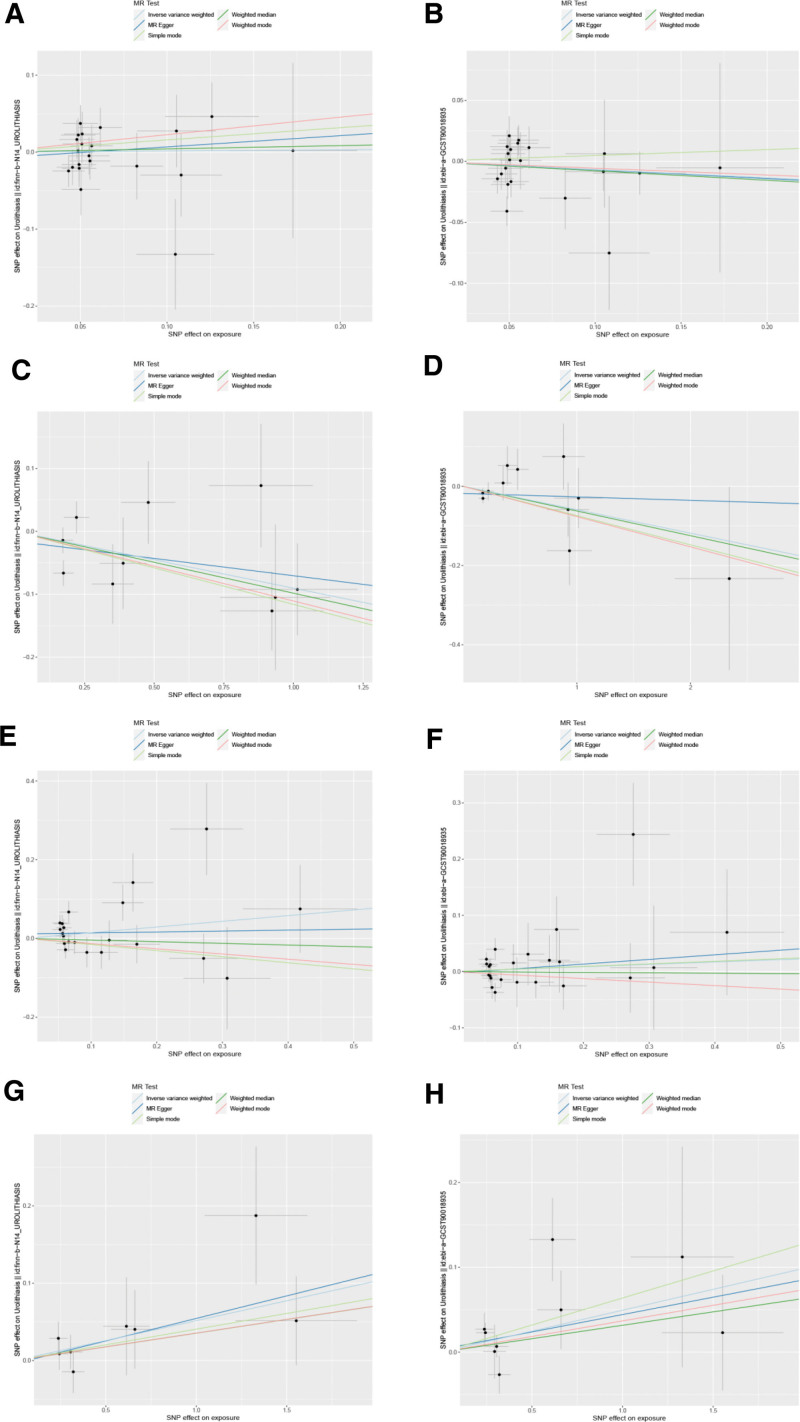
Scatterplot analysis: (A) Omeprazole–UL (test); (B) Omeprazole–UL (validation); (C) Esomeprazole–UL (test); (D) Esomeprazole–UL (validation); (E) Lansoprazole–UL (test); (F) Lansoprazole–UL (validation); (G) Rabeprazole–UL (test); (H) Rabeprazole–UL (validation). UL = urolithiasis.

Following a rigorous evaluation process, we selected suitable SNPs as IVs to fulfill 3 crucial assumptions (Fig. 1). The *F* statistic for every variant were much >10 in our study. We discovered that there was a 5% increase in the incidence of UL in the use of Rabeprazole medication (test group: OR = 1.053, 95% CI: 1.005–1.103; validation group: OR = 1.051, 95% CI: 1.003–1.101) (Figs. 2 and 3). Furthermore, we found that Esomeprazole was negatively correlated with UL in the IVW method (test group: OR = 0.914, 95% CI: 0.841–0.993; validation group: OR = 0.943, 95% CI: 0.890–0.999). The remaining 4 methods yielded similar trends, even if some *P* values did not reach statistical significance. However, no method was found to show a causal relationship between Omeprazole or Lansoprazole and UL, either in the test and validation group (Figs. 2 and 3). The SNPs features included in this study are listed in Tables S1 and S2, Supplemental Digital Content, https://links.lww.com/MD/Q682. According to the results of Cochran *Q* test and funnel plot (Fig. 4), there was no heterogeneity in the test group and the validation group. Moreover, no evidence of pleiotropy was found in the MR-Egger regression. Further analysis, including the leave-one-out analysis (Fig. 5) and the MR pleiotropy residual sum and outlier test for outliers, revealed no influential SNPs in the PPIs-UL causal association or any outliers. The results of the sensitivity analysis are presented in Table S3, Supplemental Digital Content, https://links.lww.com/MD/Q682. Figure 6 illustrates the results of the scatterplot analysis.

## 4. Discussion

This study provided the first comprehensive analysis of the impact of PPIs on UL, using summary GWAS data. Some of our findings are not consistent with previous observational studies. We discovered that there was a 5% increase in the incidence of UL in the use of Rabeprazole medication (test group: OR = 1.053, 95% CI: 1.005–1.103; validation group: OR = 1.051, 95% CI: 1.003–1.101). Furthermore, we found that Esomeprazole was negatively correlated with UL in the IVW method (test group: OR = 0.914, 95% CI: 0.841–0.993; validation group: OR = 0.943, 95% CI: 0.890–0.999).

To our knowledge, the causal relationship between PPIs and UL is currently unclear due to the lack of randomized controlled studies. Multiple studies have indicated a potential link between the use of PPIs and an elevated risk of kidney stones, with a clear dose–response relationship observed.^[7–9]^ A retrospective analysis of the Women’s Veterans Cohort, comprising 465,891 participants, highlighted a 1.25-fold increase in the likelihood of kidney stones among PPI users (95% CI, 1.19–1.33).^[8]^ However, it is important to acknowledge that this cohort was predominantly composed of young individuals, with a median age of 32 years, and was heavily skewed towards males (86%), which may have introduced a selection bias. Another study conducted by Sui et al among patients with GERD corroborated these findings, reporting a 1.46-fold increase in kidney stone risk associated with PPI use (95% CI, 1.38–1.55).^[9]^ These results are instrumental in gauging the potential risks associated with PPI exposure. However, the applicability of these findings to a broader population is limited due to the specific demographic of the study subjects. In contrast, a nationwide Korean cohort study, free from selection bias, also demonstrated a positive correlation between PPI use and the incidence of kidney stones, further reinforcing the dose-response relationship.^[7]^ Moreover, a cross-sectional study based on the National Health and Nutrition Examination Survey database also found a positive relationship between the use of PPIs and the risk of kidney stones.^[34]^ However, these studies lack information regarding the types of PPIs used, making it impossible to discern the individual effects of different PPIs medications on the formation of urinary stones. Furthermore, these studies were prone to the influence of confounding variables and reverse causation. So, we used MR analysis which could avoid this weakness to yield a relatively reasonably accurate causal conclusion.

The biological mechanism underlying the relationship between PPIs and UL remains unknown. Research indicates that the use of PPIs can increase gastric pH, potentially diminishing magnesium absorption and urinary magnesium concentrations.^[35]^ Magnesium is recognized for its role in preventing the crystallization of calcium oxalate in urine, a key factor in kidney stone formation.^[36,37]^ A comprehensive meta-analysis encompassing 9 observational studies has identified a noteworthy rise in the risk of hypomagnesaemia among individuals on PPI therapy.^[38]^ It is important to recognize that magnesium absorption is facilitated by both active and passive processes, with pH levels having no bearing on passive absorption mechanisms. Consequently, while PPIs do not invariably lead to hypomagnesaemia, those with compromised gastrointestinal absorption may be more susceptible to this condition. Conversely, existing literature has demonstrated that citrate can impede the crystallization of calcium salts in urine, and a deficiency in citrate levels can escalate the likelihood of stone formation. A detailed study of 301 nephrolithiasis patients, incorporating 24-hour urine data, revealed that exposure to PPIs significantly curtailed urinary citrate excretion without impacting urinary magnesium, pH, or other urinary minerals.^[39]^ Echoing these findings, another investigation among GERD patients established a significant association between PPI usage and diminished urinary citrate and magnesium levels.^[9]^ However, the specific mechanism needs to be further studied.

There are several advantages in our MR study. To begin with, this is the first study that, to the best of our knowledge, assesses the relationship between PPIs and UL using a 2-sample Mendelian randomization study. When MR analysis was used, potential bias such as reverse causation and confounders could be successfully reduced, strengthening the causal inference. Second, to avoid population overlap by selecting exposure and outcome data from different databases. In addition, the GWAS data of UL from 2 database were selected to validate our results. Third, a variety of methods were used for MR analysis and exclusion of heterogeneity and pleiotropic analysis. Meanwhile, our study also has some flaws and shortcomings. Due to the absence of data regarding the dosage and duration of PPIs usage, a more comprehensive analysis is not feasible. Future research endeavors could focus on filling this gap in our understanding.

## 5. Conclusion

In summary, this research aimed to evaluate the genetic correlation of PPIs with UL using MR analysis. The results of this study demonstrated a causal connection between PPIs and UL. For patients requiring antiacid therapy, esomeprazole may be a better option than Rabeprazole, especially in patients with high-risk factors for urolithiasis. Of course, this needs more research to justify it.

## Acknowledgments

We thank all of the investigators of the GWAS Catalog and IEU OpenGWAS project for sharing summary-level data on GWAS for PPIs and UL.

## Author contributions

**Conceptualization:** Jiawei Guo, Tao Zhao.

**Formal analysis:** Jiawei Guo.

**Methodology:** Jiawei Guo, Xinyu Chen.

**Supervision:** Tao Zhao.

**Visualization:** Yongqi Dou.

**Writing – original draft:** Jiawei Guo, Yongjiang Xiong.

**Writing – review & editing:** Tao Zhao.

## Supplementary Material


