# Supplementary material for: Proton pump inhibitors and the risk of urolithiasis: A Mendelian randomization study
Source: Medicine (Baltimore). 2025 Nov 21;104(47):e45646. doi: 10.1097/MD.0000000000045646 (PMC12643632; doi:10.1097/MD.0000000000045646)
Supplement: Supplementary file 1 [file medi-104-e45646-s001.docx]

**Supplementary table 1: SNPs from GWAS on PPIs and UL in Test group.**

| exposure | outcome | SNP | EA | OA | beta | se | pval | F |
| --- | --- | --- | --- | --- | --- | --- | --- | --- |
| Omeprazole | UL | rs11040831 | A | G | 0.062 | 0.012 | 7.66E-07 | 24.44 |
| Omeprazole | UL | rs115207920 | A | T | -0.108 | 0.024 | 4.58E-06 | 21.01 |
| Omeprazole | UL | rs11790051 | C | G | -0.051 | 0.010 | 9.70E-07 | 23.99 |
| Omeprazole | UL | rs13100451 | A | G | -0.056 | 0.012 | 2.17E-06 | 22.44 |
| Omeprazole | UL | rs17092943 | T | C | 0.051 | 0.011 | 4.10E-06 | 21.22 |
| Omeprazole | UL | rs28588631 | A | G | 0.050 | 0.011 | 4.95E-06 | 20.86 |
| Omeprazole | UL | rs3817459 | A | G | 0.049 | 0.010 | 6.90E-07 | 24.64 |
| Omeprazole | UL | rs384262 | G | A | -0.045 | 0.009 | 7.76E-07 | 24.42 |
| Omeprazole | UL | rs4240631 | G | A | 0.048 | 0.010 | 2.34E-06 | 22.30 |
| Omeprazole | UL | rs4422827 | T | A | 0.043 | 0.009 | 2.78E-06 | 21.96 |
| Omeprazole | UL | rs4623058 | T | A | -0.055 | 0.012 | 2.90E-06 | 21.88 |
| Omeprazole | UL | rs4676893 | A | T | 0.049 | 0.010 | 6.29E-07 | 24.82 |
| Omeprazole | UL | rs62282693 | T | A | -0.057 | 0.012 | 3.57E-06 | 21.48 |
| Omeprazole | UL | rs6464618 | T | C | -0.043 | 0.009 | 3.23E-06 | 21.68 |
| Omeprazole | UL | rs647035 | A | G | 0.049 | 0.010 | 3.55E-07 | 25.92 |
| Omeprazole | UL | rs7276225 | T | C | 0.049 | 0.010 | 1.09E-06 | 23.77 |
| Omeprazole | UL | rs75504366 | G | C | 0.126 | 0.027 | 3.26E-06 | 21.66 |
| Omeprazole | UL | rs76982774 | A | G | -0.173 | 0.037 | 3.25E-06 | 21.66 |
| Omeprazole | UL | rs78484848 | T | C | 0.083 | 0.015 | 5.51E-08 | 29.53 |
| Omeprazole | UL | rs79454971 | T | C | 0.105 | 0.023 | 3.64E-06 | 21.45 |
| Omeprazole | UL | rs79887313 | C | T | 0.105 | 0.023 | 3.20E-06 | 21.70 |
| Omeprazole | UL | rs9598273 | G | A | 0.050 | 0.010 | 9.77E-07 | 23.97 |
| Esomeprazole | UL | rs117201520 | C | G | 0.923 | 0.186 | 6.55E-07 | 24.74 |
| Esomeprazole | UL | rs117656972 | T | C | 0.935 | 0.198 | 2.34E-06 | 22.29 |
| Esomeprazole | UL | rs187013421 | G | A | 1.014 | 0.215 | 2.30E-06 | 22.33 |
| Esomeprazole | UL | rs3111476 | T | C | 0.351 | 0.075 | 3.17E-06 | 21.71 |
| Esomeprazole | UL | rs4478540 | T | A | 0.479 | 0.097 | 8.65E-07 | 24.21 |
| Esomeprazole | UL | rs55939202 | C | T | -0.174 | 0.037 | 2.92E-06 | 21.87 |
| Esomeprazole | UL | rs56070233 | T | C | 0.884 | 0.187 | 2.17E-06 | 22.44 |
| Esomeprazole | UL | rs72671454 | A | G | 0.389 | 0.084 | 4.25E-06 | 21.15 |
| Esomeprazole | UL | rs76358556 | G | A | 0.221 | 0.045 | 1.19E-06 | 23.60 |
| Esomeprazole | UL | rs7820543 | C | A | -0.172 | 0.037 | 3.93E-06 | 21.30 |
| Lansoprazole | UL | rs10995788 | A | G | 0.066 | 0.014 | 1.30E-06 | 23.42 |
| Lansoprazole | UL | rs11674978 | A | G | 0.053 | 0.012 | 4.57E-06 | 21.01 |
| Lansoprazole | UL | rs117570374 | T | C | 0.128 | 0.028 | 4.23E-06 | 21.16 |
| Lansoprazole | UL | rs117842899 | C | G | 0.148 | 0.031 | 2.43E-06 | 22.22 |
| Lansoprazole | UL | rs138655955 | T | C | 0.164 | 0.031 | 1.28E-07 | 27.89 |
| Lansoprazole | UL | rs144574721 | G | A | -0.116 | 0.025 | 3.93E-06 | 21.30 |
| Lansoprazole | UL | rs146833023 | T | C | 0.276 | 0.055 | 6.54E-07 | 24.75 |
| Lansoprazole | UL | rs150445630 | T | C | 0.418 | 0.087 | 1.47E-06 | 23.19 |
| Lansoprazole | UL | rs1555686 | G | A | -0.061 | 0.013 | 3.70E-06 | 21.42 |
| Lansoprazole | UL | rs1842467 | T | C | 0.056 | 0.012 | 3.71E-06 | 21.41 |
| Lansoprazole | UL | rs1890940 | C | T | -0.053 | 0.011 | 3.81E-06 | 21.36 |
| Lansoprazole | UL | rs2386125 | C | T | 0.057 | 0.012 | 1.00E-06 | 23.92 |
| Lansoprazole | UL | rs2523589 | T | G | 0.059 | 0.011 | 2.35E-07 | 26.72 |
| Lansoprazole | UL | rs541096318 | C | A | 0.271 | 0.052 | 2.35E-07 | 26.72 |
| Lansoprazole | UL | rs62052427 | T | G | -0.059 | 0.013 | 4.06E-06 | 21.24 |
| Lansoprazole | UL | rs696178 | T | G | 0.066 | 0.014 | 2.22E-06 | 22.39 |
| Lansoprazole | UL | rs72706312 | G | A | -0.094 | 0.020 | 4.32E-06 | 21.12 |
| Lansoprazole | UL | rs77410397 | A | G | 0.075 | 0.016 | 1.94E-06 | 22.65 |
| Lansoprazole | UL | rs7790767 | A | G | 0.307 | 0.066 | 3.55E-06 | 21.49 |
| Lansoprazole | UL | rs79807832 | C | T | 0.170 | 0.035 | 1.71E-06 | 22.90 |
| Lansoprazole | UL | rs828618 | A | G | 0.058 | 0.013 | 3.86E-06 | 21.34 |
| Rabeprazole | UL | rs118182511 | C | G | 0.661 | 0.133 | 6.16E-07 | 24.86 |
| Rabeprazole | UL | rs13098498 | T | C | 0.238 | 0.050 | 2.06E-06 | 22.54 |
| Rabeprazole | UL | rs142909537 | G | A | 1.553 | 0.336 | 3.80E-06 | 21.36 |
| Rabeprazole | UL | rs145923107 | A | G | 0.614 | 0.128 | 1.62E-06 | 23.00 |
| Rabeprazole | UL | rs187032336 | A | G | 1.330 | 0.284 | 2.86E-06 | 21.91 |
| Rabeprazole | UL | rs234121 | T | C | 0.305 | 0.066 | 3.83E-06 | 21.35 |
| Rabeprazole | UL | rs397937 | C | A | 0.320 | 0.062 | 2.19E-07 | 26.85 |
| Rabeprazole | UL | rs72950416 | A | G | 0.243 | 0.051 | 2.17E-06 | 22.43 |
| Rabeprazole | UL | rs7560777 | A | T | 0.227 | 0.050 | 4.70E-06 | 20.96 |

F=beta^2/se^2;(beta: effect size for SNP; se: standard error for SNP)

Abbreviations:EA, effect allele; OA, other allele;UL, urolithiasis.

**Supplementary table 2:SNPs from GWAS on PPIs and UL in Validation group.**

| exposure | outcome | SNP | EA | OA | beta | se | pval | F |
| --- | --- | --- | --- | --- | --- | --- | --- | --- |
| Omeprazole | UL | rs11040831 | A | G | 0.062 | 0.012 | 7.66E-07 | 24.44 |
| Omeprazole | UL | rs115207920 | A | T | -0.108 | 0.024 | 4.58E-06 | 21.01 |
| Omeprazole | UL | rs11790051 | C | G | -0.051 | 0.010 | 9.70E-07 | 23.99 |
| Omeprazole | UL | rs13100451 | A | G | -0.056 | 0.012 | 2.17E-06 | 22.44 |
| Omeprazole | UL | rs17092943 | T | C | 0.051 | 0.011 | 4.10E-06 | 21.22 |
| Omeprazole | UL | rs28588631 | A | G | 0.050 | 0.011 | 4.95E-06 | 20.86 |
| Omeprazole | UL | rs3817459 | A | G | 0.049 | 0.010 | 6.90E-07 | 24.64 |
| Omeprazole | UL | rs384262 | G | A | -0.045 | 0.009 | 7.76E-07 | 24.42 |
| Omeprazole | UL | rs4240631 | G | A | 0.048 | 0.010 | 2.34E-06 | 22.30 |
| Omeprazole | UL | rs4422827 | T | A | 0.043 | 0.009 | 2.78E-06 | 21.96 |
| Omeprazole | UL | rs4623058 | T | A | -0.055 | 0.012 | 2.90E-06 | 21.88 |
| Omeprazole | UL | rs4676893 | A | T | 0.049 | 0.010 | 6.29E-07 | 24.82 |
| Omeprazole | UL | rs62282693 | T | A | -0.057 | 0.012 | 3.57E-06 | 21.48 |
| Omeprazole | UL | rs6464618 | T | C | -0.043 | 0.009 | 3.23E-06 | 21.68 |
| Omeprazole | UL | rs647035 | A | G | 0.049 | 0.010 | 3.55E-07 | 25.92 |
| Omeprazole | UL | rs7276225 | T | C | 0.049 | 0.010 | 1.09E-06 | 23.77 |
| Omeprazole | UL | rs75504366 | G | C | 0.126 | 0.027 | 3.26E-06 | 21.66 |
| Omeprazole | UL | rs76982774 | A | G | -0.173 | 0.037 | 3.25E-06 | 21.66 |
| Omeprazole | UL | rs78484848 | T | C | 0.083 | 0.015 | 5.51E-08 | 29.53 |
| Omeprazole | UL | rs79454971 | T | C | 0.105 | 0.023 | 3.64E-06 | 21.45 |
| Omeprazole | UL | rs79887313 | C | T | 0.105 | 0.023 | 3.20E-06 | 21.70 |
| Omeprazole | UL | rs9598273 | G | A | 0.050 | 0.010 | 9.77E-07 | 23.97 |
| Esomeprazole | UL | rs117201520 | C | G | 0.923 | 0.186 | 6.55E-07 | 24.74 |
| Esomeprazole | UL | rs117656972 | T | C | 0.935 | 0.198 | 2.34E-06 | 22.29 |
| Esomeprazole | UL | rs139294193 | G | C | 2.340 | 0.478 | 9.79E-07 | 23.97 |
| Esomeprazole | UL | rs187013421 | G | A | 1.014 | 0.215 | 2.30E-06 | 22.33 |
| Esomeprazole | UL | rs3111476 | T | C | 0.351 | 0.075 | 3.17E-06 | 21.71 |
| Esomeprazole | UL | rs4478540 | T | A | 0.479 | 0.097 | 8.65E-07 | 24.21 |
| Esomeprazole | UL | rs55939202 | C | T | -0.174 | 0.037 | 2.92E-06 | 21.87 |
| Esomeprazole | UL | rs56070233 | T | C | 0.884 | 0.187 | 2.17E-06 | 22.44 |
| Esomeprazole | UL | rs72671454 | A | G | 0.389 | 0.084 | 4.25E-06 | 21.15 |
| Esomeprazole | UL | rs76358556 | G | A | 0.221 | 0.045 | 1.19E-06 | 23.60 |
| Esomeprazole | UL | rs7820543 | C | A | -0.172 | 0.037 | 3.93E-06 | 21.30 |
| Lansoprazole | UL | rs10415242 | A | G | 0.099 | 0.021 | 1.80E-06 | 22.79 |
| Lansoprazole | UL | rs10995788 | A | G | 0.066 | 0.014 | 1.30E-06 | 23.42 |
| Lansoprazole | UL | rs11674978 | A | G | 0.053 | 0.012 | 4.57E-06 | 21.01 |
| Lansoprazole | UL | rs117570374 | T | C | 0.128 | 0.028 | 4.23E-06 | 21.16 |
| Lansoprazole | UL | rs117842899 | C | G | 0.148 | 0.031 | 2.43E-06 | 22.22 |
| Lansoprazole | UL | rs138655955 | T | C | 0.164 | 0.031 | 1.28E-07 | 27.89 |
| Lansoprazole | UL | rs144574721 | G | A | -0.116 | 0.025 | 3.93E-06 | 21.30 |
| Lansoprazole | UL | rs146833023 | T | C | 0.276 | 0.055 | 6.54E-07 | 24.75 |
| Lansoprazole | UL | rs150445630 | T | C | 0.418 | 0.087 | 1.47E-06 | 23.19 |
| Lansoprazole | UL | rs1555686 | G | A | -0.061 | 0.013 | 3.70E-06 | 21.42 |
| Lansoprazole | UL | rs1842467 | T | C | 0.056 | 0.012 | 3.71E-06 | 21.41 |
| Lansoprazole | UL | rs1890940 | C | T | -0.053 | 0.011 | 3.81E-06 | 21.36 |
| Lansoprazole | UL | rs2386125 | C | T | 0.057 | 0.012 | 1.00E-06 | 23.92 |
| Lansoprazole | UL | rs2523589 | T | G | 0.059 | 0.011 | 2.35E-07 | 26.72 |
| Lansoprazole | UL | rs541096318 | C | A | 0.271 | 0.052 | 2.35E-07 | 26.72 |
| Lansoprazole | UL | rs569327140 | C | T | -0.159 | 0.034 | 3.24E-06 | 21.67 |
| Lansoprazole | UL | rs62052427 | T | G | -0.059 | 0.013 | 4.06E-06 | 21.24 |
| Lansoprazole | UL | rs696178 | T | G | 0.066 | 0.014 | 2.22E-06 | 22.39 |
| Lansoprazole | UL | rs72706312 | G | A | -0.094 | 0.020 | 4.32E-06 | 21.12 |
| Lansoprazole | UL | rs77410397 | A | G | 0.075 | 0.016 | 1.94E-06 | 22.65 |
| Lansoprazole | UL | rs7790767 | A | G | 0.307 | 0.066 | 3.55E-06 | 21.49 |
| Lansoprazole | UL | rs79807832 | C | T | 0.170 | 0.035 | 1.71E-06 | 22.90 |
| Lansoprazole | UL | rs828618 | A | G | 0.058 | 0.013 | 3.86E-06 | 21.34 |
| Rabeprazole | UL | rs10756389 | A | G | -0.294 | 0.064 | 3.69E-06 | 21.42 |
| Rabeprazole | UL | rs118182511 | C | G | 0.661 | 0.133 | 6.16E-07 | 24.86 |
| Rabeprazole | UL | rs13098498 | T | C | 0.238 | 0.050 | 2.06E-06 | 22.54 |
| Rabeprazole | UL | rs142909537 | G | A | 1.553 | 0.336 | 3.80E-06 | 21.36 |
| Rabeprazole | UL | rs145923107 | A | G | 0.614 | 0.128 | 1.62E-06 | 23.00 |
| Rabeprazole | UL | rs187032336 | A | G | 1.330 | 0.284 | 2.86E-06 | 21.91 |
| Rabeprazole | UL | rs234121 | T | C | 0.305 | 0.066 | 3.83E-06 | 21.35 |
| Rabeprazole | UL | rs397937 | C | A | 0.320 | 0.062 | 2.19E-07 | 26.85 |
| Rabeprazole | UL | rs72950416 | A | G | 0.243 | 0.051 | 2.17E-06 | 22.43 |
| Rabeprazole | UL | rs7560777 | A | T | 0.227 | 0.050 | 4.70E-06 | 20.96 |

F=beta^2/se^2;(beta: effect size for SNP; se: standard error for SNP)

Abbreviations:EA, effect allele; OA, other allele;UL, urolithiasis.

**Supplementary table 3: Results of sensitivity analysis**

| Exposure | Outcome | MR-Egger | | Cochrane's Q test | | Rucker's Q test | |
| --- | --- | --- | --- | --- | --- | --- | --- |
|  |  | Egger Intercept | Pintercept | QIVW-Heter | PIVW-Heter | QEgger-Heter | PEgger-Heter |
| Omeprazole | UL(Test) | -0.007853923 | 0.708480597 | 17.53767301 | 0.617834834 | 17.39360666 | 0.563220642 |
| Esomeprazole | UL(Test) | -0.015482965 | 0.535510873 | 14.28274504 | 0.11261693 | 13.57173219 | 0.093633914 |
| Lansoprazole | UL(Test) | 0.011498109 | 0.487734103 | 30.96792072 | 0.055615382 | 30.172614 | 0.049642326 |
| Rabeprazole | UL(Test) | -0.003835587 | 0.832633072 | 4.011145713 | 0.778492747 | 3.962430483 | 0.681760747 |
| Omeprazole | UL(Validation) | 0.000280785 | 0.981942354 | 27.71053315 | 0.116430697 | 27.70976609 | 0.089175631 |
| Esomeprazole | UL(Validation) | -0.017978131 | 0.213700225 | 12.11550299 | 0.27740071 | 10.10523907 | 0.342032926 |
| Lansoprazole | UL(Validation) | -0.00318266 | 0.780338869 | 29.51356634 | 0.13077429 | 29.40184426 | 0.104683341 |
| Rabeprazole | UL(Validation) | 0.003282462 | 0.86564796 | 11.03067876 | 0.19996695 | 10.98235048 | 0.139390739 |
